# Persistent Air Leak Due to Chest Drain Malposition

**DOI:** 10.7759/cureus.49255

**Published:** 2023-11-22

**Authors:** Ghaith Qsous, Vipin Zamvar

**Affiliations:** 1 Cardiothoracic Surgery, Royal Infirmary Hospital of Edinburgh, Edinburgh, GBR

**Keywords:** thoracic surgery, malposition, chest tube, persistent air leak, spontaneous pneumothorax

## Abstract

Persistent or prolonged air leak (PAL) is one of the common complications that may happen after many procedures in thoracic surgery. The treatment may change based on the cause, and accordingly, the understanding and awareness of the causes and the exclusion of the rare causes are very important in the treatment of this condition. Here, we present an unusual case with PAL due to chest drain malposition with intraparenchymal insertion in an elderly patient who presented initially with a secondary spontaneous pneumothorax (SSP).

## Introduction

Spontaneous pneumothorax (SP) is a common condition that we frequently meet in our practice as thoracic surgeons, with annual incidences of admissions of approximately 14% [1]. These can be divided into primary or secondary spontaneous pneumothoraces (PSP/SSP). Primary spontaneous pneumothorax (PSP) happens in the absence of preexisting lung pathology, while SSP is associated with underlying pulmonary disease. This is most often chronic obstructive pulmonary disease (COPD) or interstitial lung disease (ILD) [2].

One of the most common consequences of SSP is a persistent air leak (PAL). This is defined as an air leak that persists for greater than 5-7 days. There are multiple causes, including the presence of an alveolar pleural fistula or a bronchial pleural fistula. PALs are associated with higher morbidity, chest infection, increased length of stay in the hospital, and increased period of chest drain. Accordingly, the recognition of the cause of the PAL and timely management are essential [3,4].

We describe a case of an SSP on the background of advanced lung fibrosis, which was complicated by a PAL due to chest drain malposition. Our case highlights the importance of considering this cause in the differential diagnoses of persistent air leaks.

## Case presentation

A 78-year-old male patient, with a background of prior tobacco smoking, high body mass index (BMI), historic asbestos exposure, and advanced pulmonary fibrosis, presented to his local hospital complaining of shortness of breath. He previously had a left video-assisted thoracoscopic (VATS) bullectomy and talc pleurodesis for a secondary spontaneous pneumothorax.

On clinical inquiry, the patient mentioned that he had experienced a productive cough for a fortnight and that this had been treated with antibiotics. This had failed to improve and was still present on arrival at the hospital with the pneumothorax. It is worth mentioning that the patient’s history was similar to that which had preceded his first pneumothorax in 2019.

On examination, the patient appeared tired with significant dyspnea and tachypnea. His oximeter oxygen saturation was 71% on room air and improved to 82% on 19 L of oxygen. His respiratory rate was 36 breaths per minute, alongside a sinus tachycardia of 110 beats per minute and blood pressure of 193/88 mmHg. He remained afebrile. He had absent breath sounds on the right side.

**Figure 1:**
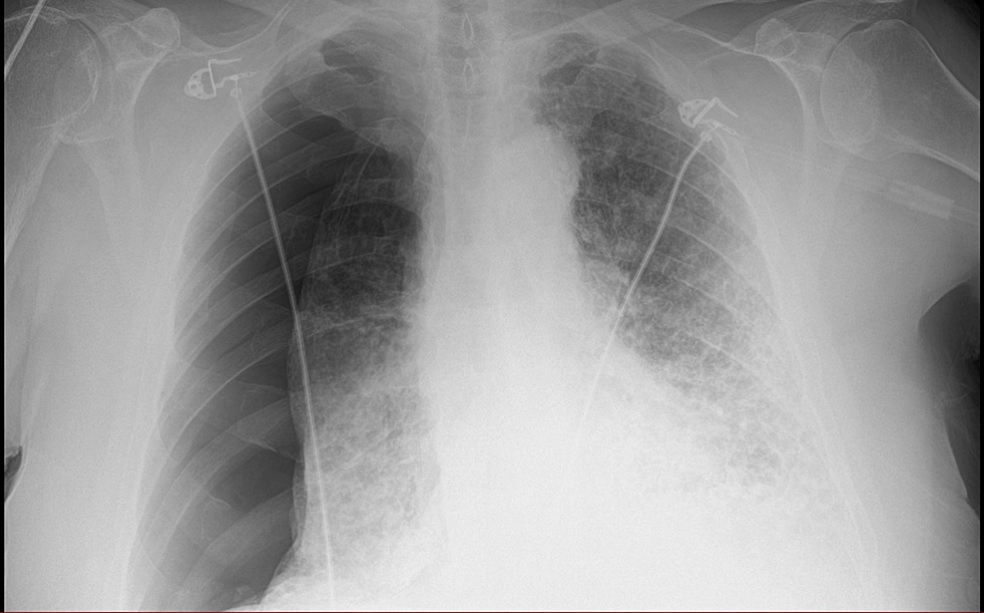
Right-sided pneumothorax with a collapsed lung. Anterior-posterior (AP), portable chest X-ray.

**Figure 2:**
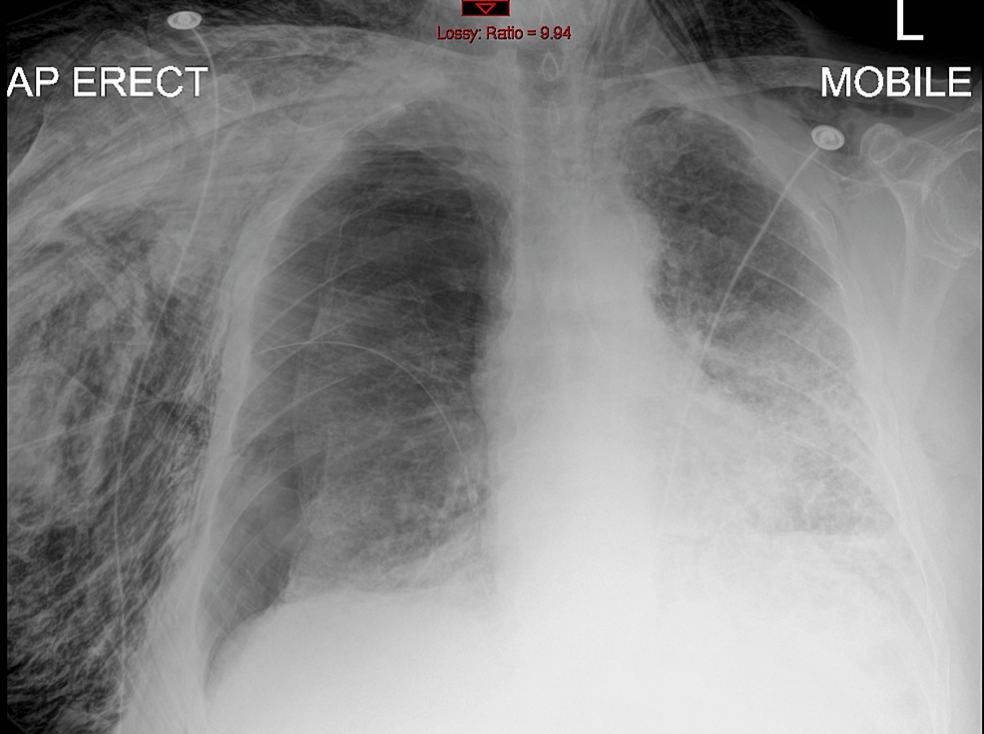
Decrease in the size of the right-sided pneumothorax, with concomitant increase in subcutaneous emphysema. Anterior-posterior (AP), chest X-ray with chest drain on the right side.

Anterior-posterior, portable chest X-ray showed a large, right-sided pneumothorax (Figure 1), and accordingly, the Seldinger technique was used for chest drain insertion. After drain insertion, the patient’s condition improved. The follow-up chest X-ray after two days showed a decrease in the size of the pneumothorax and significant subcutaneous emphysema (Figure 2). Accordingly, the decision was made to transfer the patient to a tertiary hospital where thoracic surgery expertise was available.

**Figure 3:**
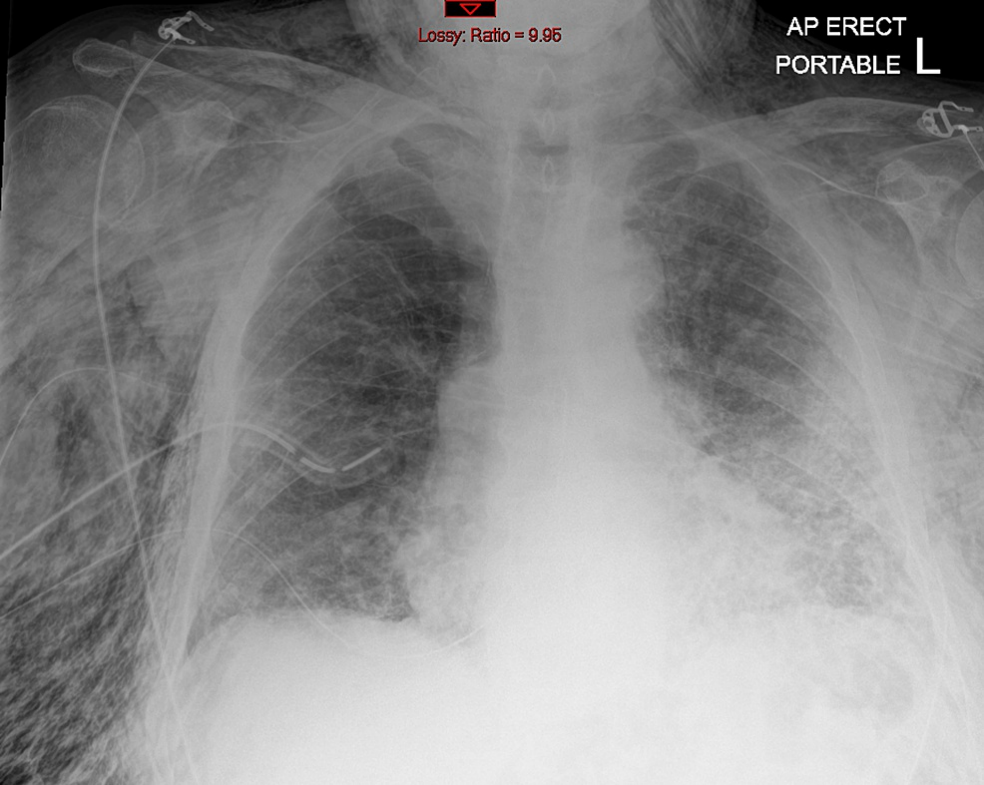
Second chest drain inserted with a subsequent decrease in the volume of the pneumothorax. Anterior-posterior (AP), portable chest X-ray.

The patient was admitted to the high-dependency unit (HDU) for close observation. On arrival, he was on 15 L of oxygen with an oxygen saturation of 88%. He had significant subcutaneous emphysema bilaterally, extending to his face proximally and his abdomen distally. As a result, a second large-bore chest drain was inserted (Figure 3), and two subcutaneous drains were inserted. All drains were on a suction of 1.5-2.5 kPa. The surgical drain was inserted under local anesthesia and an aseptic technique with the identification of the safe triangle and the aim to go into the fifth intercostal space. After cutting the skin, the layers were dissected with a surgical instrument (Roberts) until the pleura was reached and opened with the instrument. Taking into consideration the massive subcutaneous emphysema and the high body mass index (BMI), there was a technical difficulty in the procedure. The intercostal drains bubbled on coughing.

Over the next few days, the patient’s condition improved, and he was transferred to the ward on 4 L of oxygen and a saturation of up to 92%. Taking into consideration his current condition and the high risk of general anesthesia and one-lung ventilation, the decision was made to continue conservative management. At that time, both the patient’s drains continued to show air leaks, and he continued to demonstrate significant, widespread subcutaneous emphysema.

**Figure 4:**
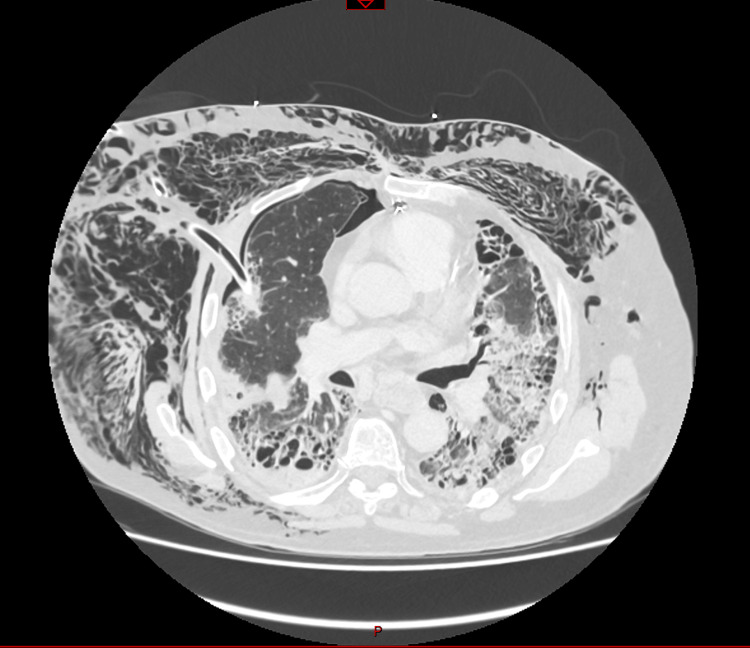
Chest drain malposition with penetration of the right lung: significant subcutaneous emphysema. Chest CT scan with contrast.

**Figure 5:**
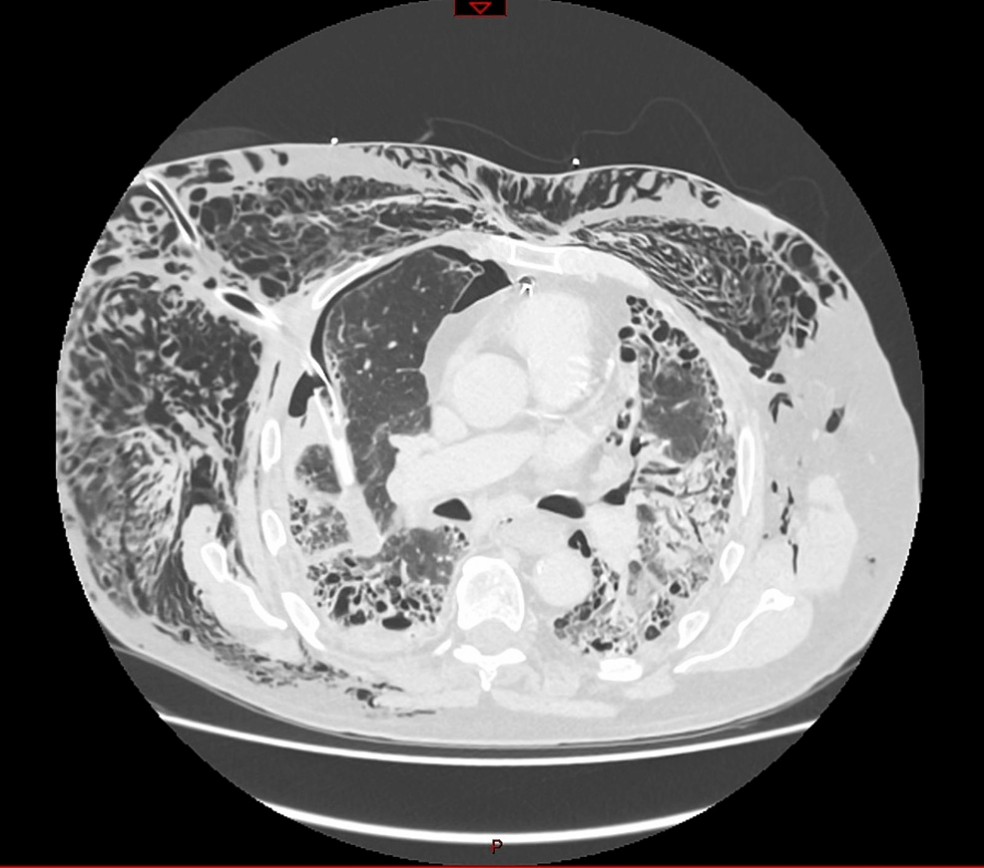
Chest drain intraparenchymal of the right lung. Chest CT scan with contrast. Chest drain penetrating the right lung.

However, shortly after returning to the ward, the patient demonstrated an increasing oxygen requirement. Given the persistent subcutaneous emphysema, a chest CT scan was performed and reported a suspicion of chest drain malposition (Figures 4, 5).

**Figure 6:**
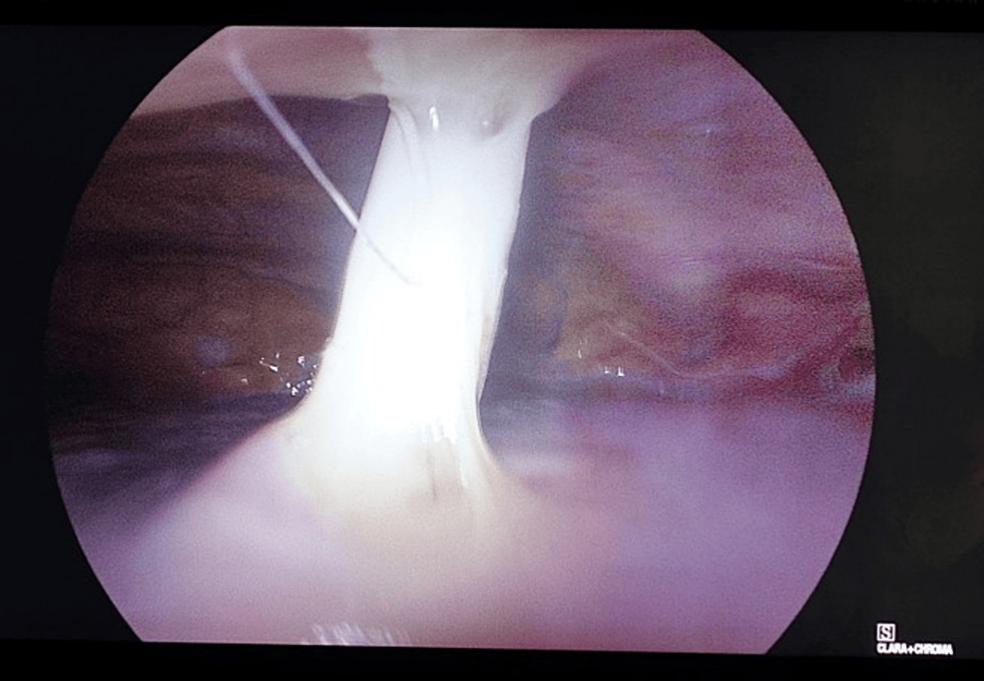
Drain in the lung. Picture from the video-assisted thoracoscopic (VATS) surgery.

**Figure 7:**
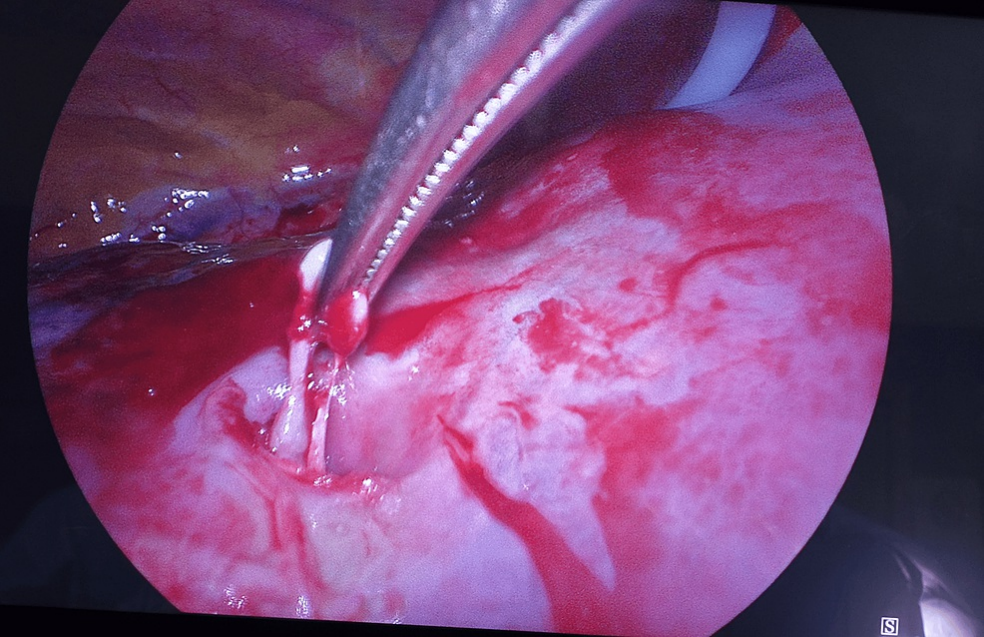
Hole in the lung. Picture from the video-assisted thoracoscopic (VATS) surgery. The surgical instrument pointing to the hole in the right lung, which was done by the chest drain.

Accordingly, after discussion with the anesthetic team and the patient, the decision was made to proceed to VATS exploration for drain removal and talc pleurodesis. Intraoperatively, the large-bore chest drain was found to be penetrating the lung (Figures 6, 7) It was therefore removed without complication, leaving a lesion of approximately 1.5 × 1.5 cm. Due to the advanced lung fibrosis and the fragility of the lung tissue, it was then decided to finish the operation with talc pleurodesis and the insertion of a new chest drain.

Postoperatively, the patient’s condition significantly improved, and his oxygen requirements decreased. The air leak stopped, and his subcutaneous emphysema significantly decreased.

The drains were removed after a few days, and the patient then underwent respiratory rehabilitation and physiotherapy. He was discharged in a stable clinical condition, without an oxygen requirement and a saturation of 90% on room air.

## Discussion

A pneumothorax with a persistent air leak is a condition that thoracic surgeons frequently encounter. It is known to increase patient morbidity by necessitating prolonged drain insertion and prolonged length of hospital admission and causing a higher risk and incidence of infection. The management of PAL can be divided into surgical and nonsurgical, depending on the etiology and patient’s condition. Identifying the cause of PAL can facilitate and optimize the management of this condition [4,5].

Chest drain insertion is considered the initial treatment for pneumothorax and PAL, yet it is clear that this intervention can carry some risks and complications. The lung is the most commonly injured organ during chest drain insertion, and drain malposition is one of the most common complications related to drain insertion. An intraparenchymal location of the chest drain is the most common site of a malpositioned chest drain. Patients with underlying lung disease or significant pleural adhesions are at an increased risk for lung injury and malposition [6,7].

Remerand et al. performed a prospective study on 122 chest drains percutaneously inserted in 75 consecutive critically ill patients. They found that 10 chest drains were inserted intraparenchymally (9%). The only predicting factor associated with the risk of malposition was the use of a trocar for the percutaneous insertion of the chest tube (P = 0.032) [8].

A diagnosis of an intraparenchymal malposition of a chest drain can be delayed or missed because radiographic evidence is absent and many patients do not exhibit signs or symptoms, especially those with underlying pulmonary disease. Sargi et al. [9] and Santana-Cabrera et al. [10] both reported similar cases wherein patients with pneumothoraces were treated with chest drain insertion and follow-up chest X-rays failed to show any abnormal findings. However, subsequent chest CT scans did show that there was intraparenchymal chest drain malposition. Accordingly, a chest CT scan is important as a follow-up if the patient has PAL after drain insertion.

Chest drain malposition with intraparenchymal location can become complicated with PAL and diffuse subcutaneous emphysema. As a result, the patient’s condition can deteriorate and increase the risk of morbidity and mortality. An appropriate imaging technique should be chosen for the early diagnosis of drain malposition. Definitive treatment includes drain replacement and treating the PAL based on the patient’s condition.

## Conclusions

Chest drain malposition with intraparenchymal insertion should be considered as a rare but clinically important cause of persistent air leak. Chest CT scan is the appropriate imaging modality to diagnose drain malposition. It also provides useful information for subsequent surgery that may be required.
